# Supplementary material for: Mapping developmental patterns of intrinsic timescale
Source: bioRxiv. 2026 Apr 9:2026.04.08.717312. Preprint. [Version 1] doi: 10.64898/2026.04.08.717312 (PMC13081997; doi:10.64898/2026.04.08.717312)
Supplement: Supplement 1 [file NIHPP2026.04.08.717312v1-supplement-1.pdf]

bioRxiv preprint doi: https://doi.org/10.64898/2026.04.08.717312; this version posted April 9, 2026. The copyright holder for this preprint (which was not certified by peer review) is the author/funder, who has granted bioRxiv a license to display the preprint in perpetuity. It is made available under aCC-BY 4.0 International license.

Supplementary figures

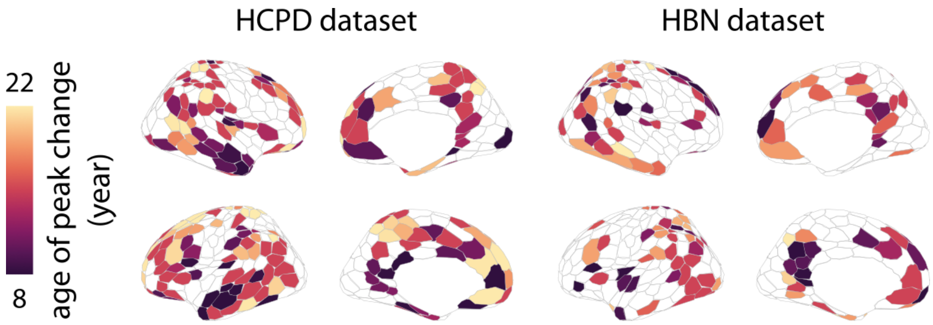

Figure S1. Similar age of peak change in intrinsic timescale across two developmental cohorts | Derivatives of the intrinsic timescale regional developmental patterns (**Figure 3d** and **Figure 4d**) were calculated as finite differences between consecutive time points to estimate the age at which timescale development demonstrated maximal change. Specifically, the age of peak change was estimated as the age where the derivative was greatest in absolute magnitude.

bioRxiv preprint doi: https://doi.org/10.64898/2026.04.08.717312; this version posted April 9, 2026. The copyright holder for this preprint (which was not certified by peer review) is the author/funder, who has granted bioRxiv a license to display the preprint in perpetuity. It is made available under aCC-BY 4.0 International license.

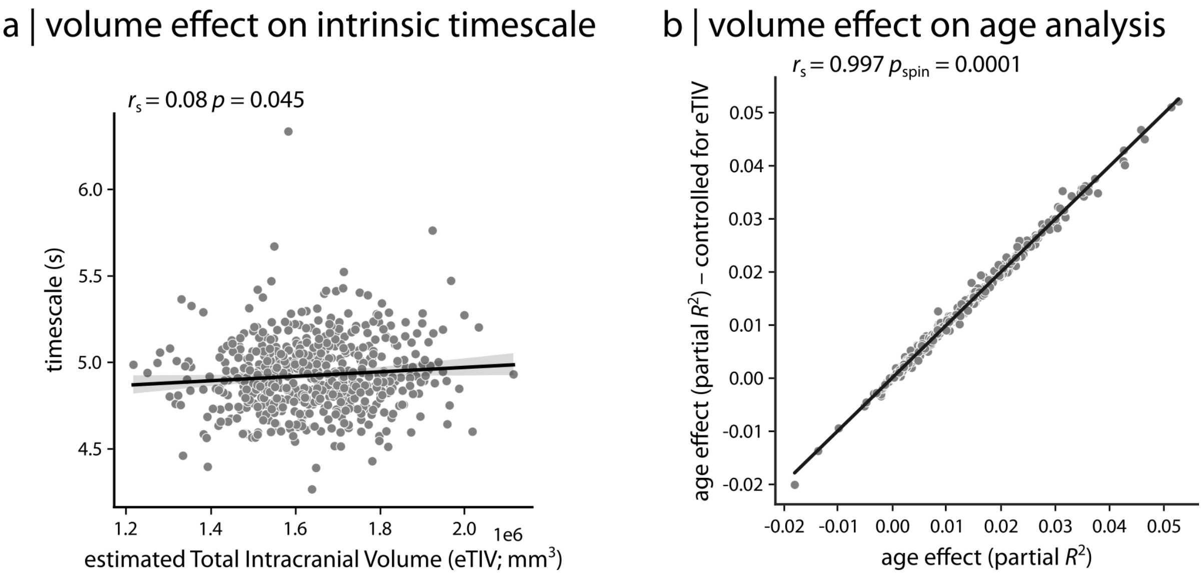

Figure S2. Effects of total brain volume on intrinsic timescale and its developmental patterns | (a) To ensure that findings were independent from changes in brain size during development, we directly examined the relationship between estimated Total Intracranial Volume (eTIV) and intrinsic timescale in the HCPD dataset and found a weak association between the two. Each data point in the scatter plot represents an individual participant. **(b)** To assess whether developmental patterns of intrinsic timescale were independent from changes in brain size, we repeated the age analysis (i.e., GAMs) while controlling for eTIV as a model covariate. The age effects (i.e., partial R^2) were consistent with the original analysis. Each data point in the scatter plot represents a brain region from the Schaefer 400 atlas. r_s denotes Spearman's rank correlation coefficient. Linear regression lines are added for visualization purposes only.

bioRxiv preprint doi: https://doi.org/10.64898/2026.04.08.717312; this version posted April 9, 2026. The copyright holder for this preprint (which was not certified by peer review) is the author/funder, who has granted bioRxiv a license to display the preprint in perpetuity. It is made available under aCC-BY 4.0 International license.

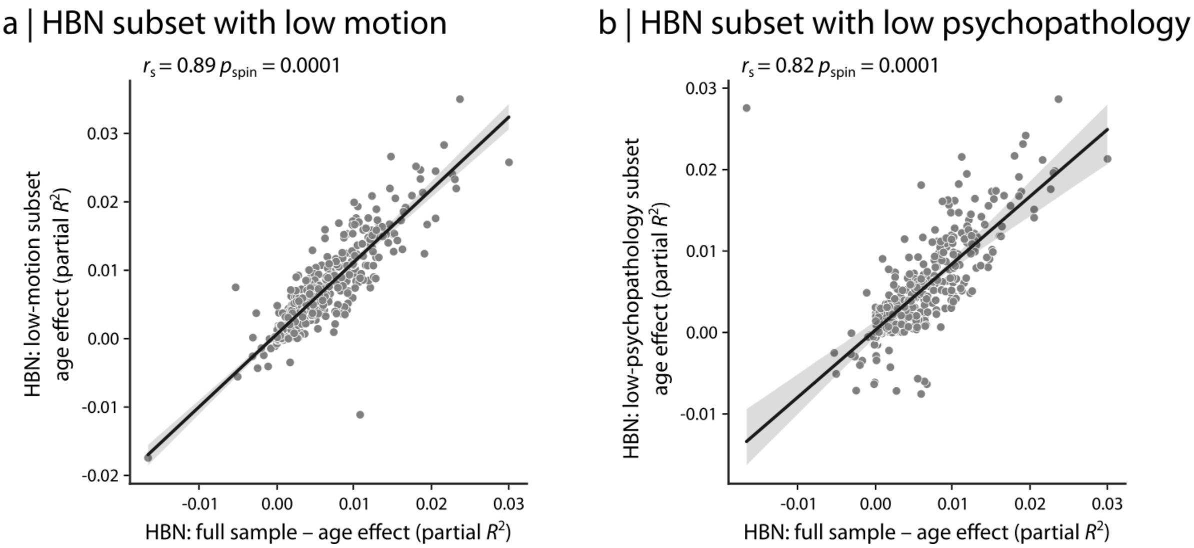

Figure S3. Consistent findings in two subsets of the HBN dataset | Given that HBN is a more heterogeneous sample compared to HCPD and includes help-seeking individuals with higher levels of psychopathology, we repeated all the analyses using two subsets of HBN individuals (each with N=600; this approximately matches the HCPD sample size) with **(a)** low motion and **(b)** low psychopathology (i.e., *p*-factor). In both cases, the developmental patterns in intrinsic timescale (i.e., partial R^2) were consistent with the full HBN sample. Each data point in the scatter plot represents a brain region from the Schaefer 400 atlas. r_s denotes Spearman's rank correlation coefficient. Linear regression lines are added for visualization purposes only.

bioRxiv preprint doi: https://doi.org/10.64898/2026.04.08.717312; this version posted April 9, 2026. The copyright holder for this preprint (which was not certified by peer review) is the author/funder, who has granted bioRxiv a license to display the preprint in perpetuity. It is made available under aCC-BY 4.0 International license.

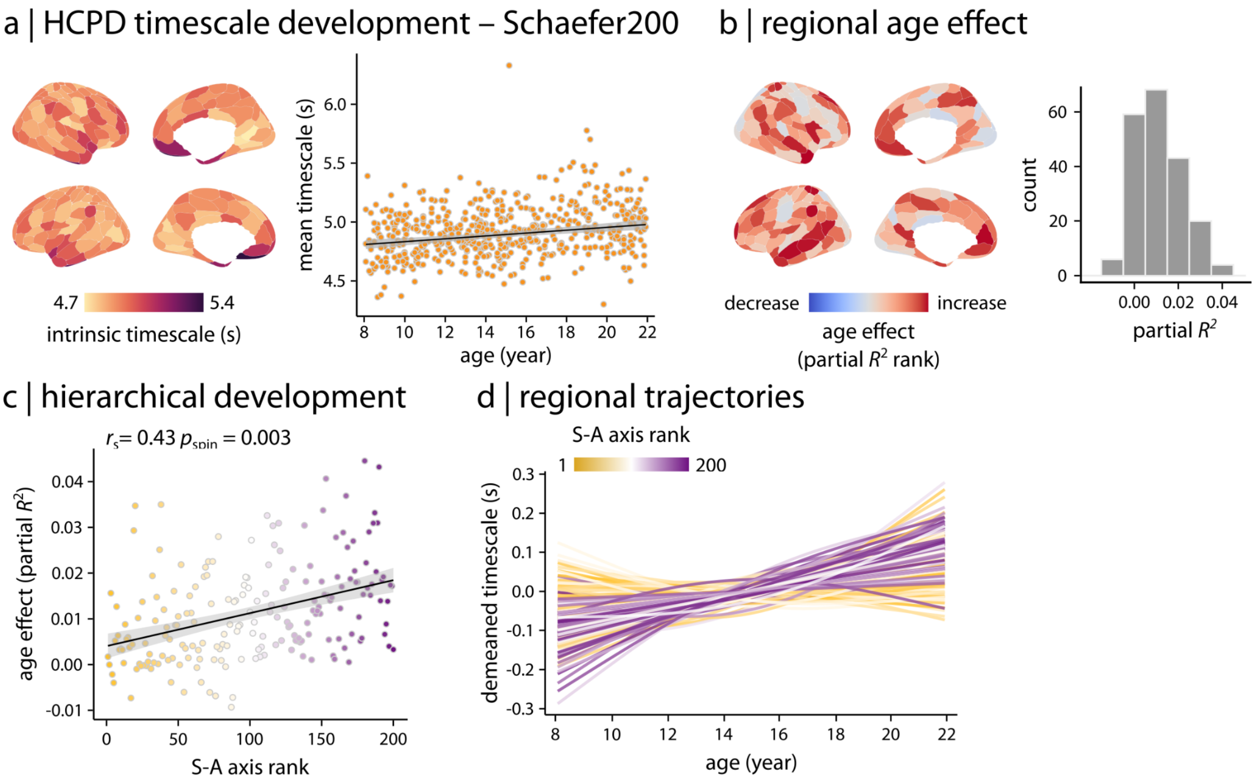

Figure S4. Consistent findings using the Schaefer 200 atlas | Developmental patterns of intrinsic timescale were replicated in the Schaefer 200 atlas in HCPD. **(a)** Consistent with the finding with the Schaefer 400 atlas (**Figure 2**), GAM results demonstrated that average timescale increases during development in youth. **(b)** Region-wise GAMs identified heterogeneous age effects (i.e., partial R^2) across the cortex. **(c)** Age effects on intrinsic timescale were hierarchically organized along the S–A axis. Significance of the association between age effects and S–A axis rank was assessed using 10,000 spin tests. r_s denotes Spearman's rank correlation coefficient. Linear regression line is added for visualization purposes only. **(d)** Regional trajectories of developmental changes in intrinsic timescale were obtained from region-wise GAM results (as shown in panel **b**). Each line corresponds to the model fit of each cortical region and is colored based on the region's rank along the S–A axis. Similar to the findings with the Schaefer 400 atlas, the results demonstrate that intrinsic timescale in association regions increases during development in youth while it remains relatively stable in sensorimotor regions.

bioRxiv preprint doi: https://doi.org/10.64898/2026.04.08.717312; this version posted April 9, 2026. The copyright holder for this preprint (which was not certified by peer review) is the author/funder, who has granted bioRxiv a license to display the preprint in perpetuity. It is made available under aCC-BY 4.0 International license.

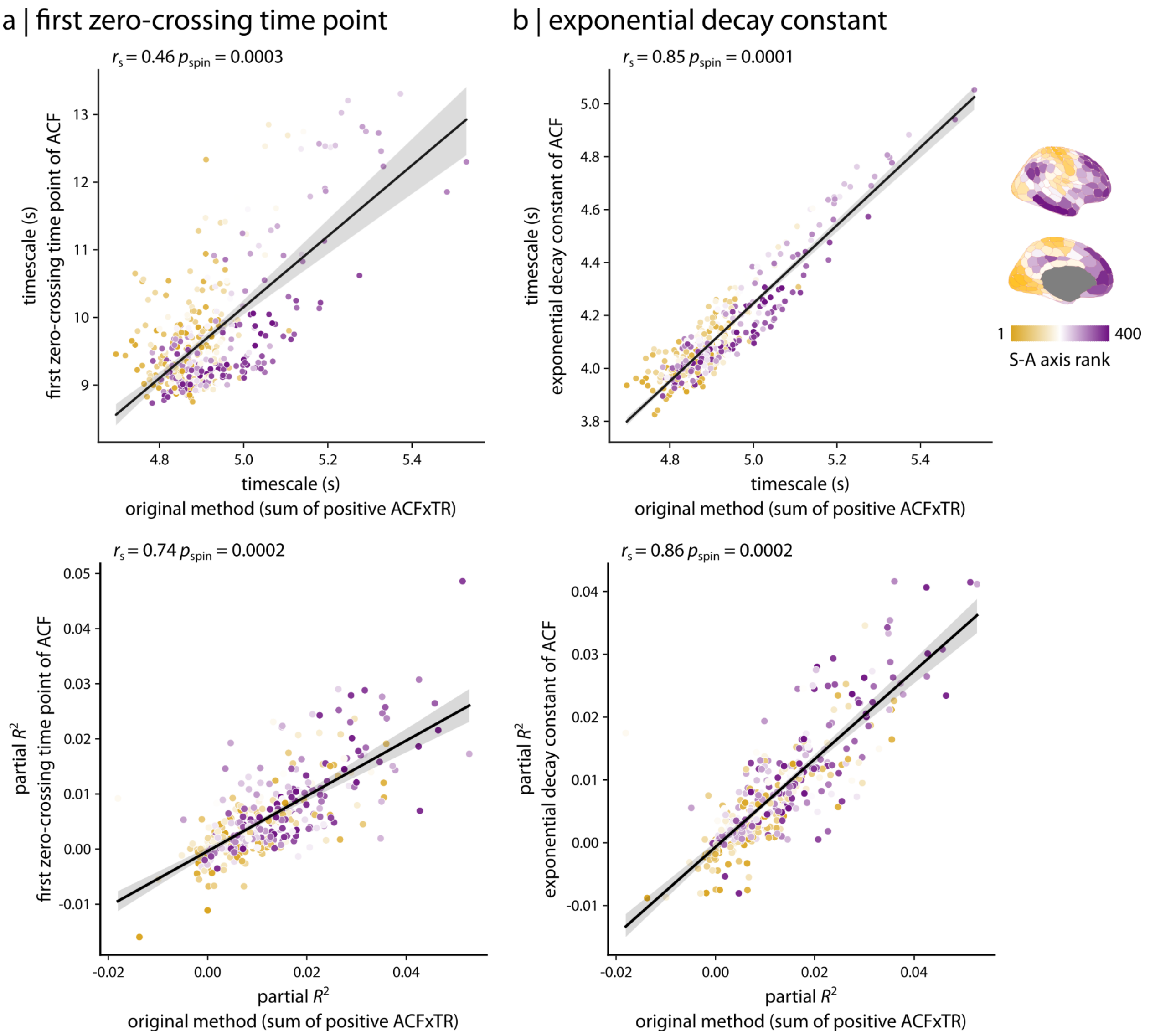

Figure S5. Convergent results with methodological sensitivity analysis in quantifying intrinsic timescale | To test whether intrinsic timescale estimates were sensitive to methodological choices, we quantified the intrinsic timescale in HCPD using two other approaches. **(a)** The intrinsic timescale was quantified as the first zero-crossing time point of the fMRI time series autocorrelation functions (ACF). **(b)** The intrinsic timescale was quantified as the exponential decay constant from an exponential fit to ACF. Both approaches generated intrinsic timescale maps consistent with the original method. Additionally, the developmental patterns were consistent with the original analysis. Each data point in the scatter plot represents a brain region from the Schaefer 400 atlas. The data points are colored based on their rankings on the S–A axis. r_s denotes Spearman's rank correlation coefficient. Linear regression lines are added for visualization purposes only.

bioRxiv preprint doi: https://doi.org/10.64898/2026.04.08.717312; this version posted April 9, 2026. The copyright holder for this preprint (which was not certified by peer review) is the author/funder, who has granted bioRxiv a license to display the preprint in perpetuity. It is made available under aCC-BY 4.0 International license.

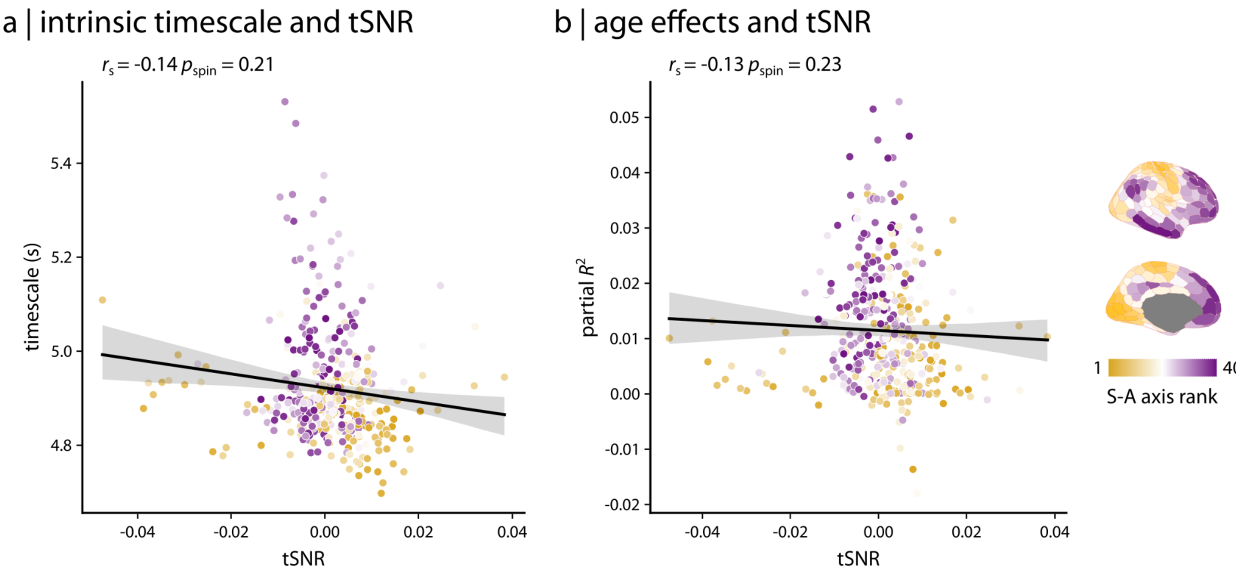

Figure S6. Findings were independent from temporal signal-to-noise ratio (tSNR) | To test whether the findings were sensitive to signal-to-noise ratio (SNR) of the fMRI signal, we estimated temporal SNR (tSNR) as the ratio of the time-series mean to standard deviation for each region and participant in HCPD. Neither **(a)** the intrinsic timescale nor **(b)** the age effects (partial R^2) were significantly associated with tSNR. Each data point in the scatter plot represents a brain region from the Schaefer 400 atlas. The data points are colored based on their rankings on the S–A axis. r_s denotes Spearman's rank correlation coefficient. Linear regression lines are added for visualization purposes only.
